# Propensity score-matching analysis to compare clinical outcomes of endoscopic submucosal dissection for early gastric cancer in the postoperative and non-operative stomachs

**DOI:** 10.1186/s12876-018-0855-2

**Published:** 2018-08-06

**Authors:** Mitsuru Esaki, Sho Suzuki, Yasuyo Hayashi, Azusa Yokoyama, Shuichi Abe, Taizo Hosokawa, Shinichi Tsuruta, Yosuke Minoda, Yoshitaka Hata, Haruei Ogino, Hirotada Akiho, Eikichi Ihara, Yoshihiro Ogawa

**Affiliations:** 1 Division of Gastroenterology and Hepatology, Department of Medicine, Nihon University School of Medicine, 1-6 Kanda-Surugadai, Chiyoda-ku, Tokyo, 101-8309 Japan; 2 Department of Gastroenterology, Kitakyushu Municipal Medical Center, 2-1-1 Bashaku, Kokurakita-ku, Kitakyushu, Fukuoka, 802-0077 Japan; 3 Department of Medicine and Bioregulatory Science, Graduate School of Medical Sciences, Kyushu University, 3-1-1 Maidashi, Higashi-ku, Fukuoka, 812-8582 Japan; 4 Department of Anatomic Pathology, Graduate School of Medical Sciences Kyushu University, 3-1-1 Maidashi, Higashi-ku, Fukuoka, 812-8582 Japan

**Keywords:** Endoscopic submucosal dissection, Postoperative stomach, Gastric cancer

## Abstract

**Background:**

Endoscopic submucosal dissection (ESD) of the postoperative stomach (ESD-P) for early gastric cancer (EGC) is considered a technically difficult procedure. However, it is difficult to compare the outcomes of ESD-P and ESD of the non-operative stomach (ESD-N) because their baseline characteristics are different. Therefore, we aimed to compare the technical outcomes of ESD-P with those of ESD-N using a propensity score-matching analysis to compensate for the differences.

**Methods:**

The chart records of 1046 patients with EGC who were treated with ESD between January 2004 and July 2016 at Kitakyushu Municipal Medical Center in Japan were reviewed in this retrospective study. Multivariate analyses and propensity score-matching were performed for age, sex, lesion location, lesion size, tumor invasion, tumor size, ulcer (scar), and operator skill. The primary outcome was procedure time. Secondary outcomes were percentages of en bloc, complete, and curative resections, and percentages of adverse events, which were evaluated between the two groups.

**Results:**

Forty-one patients were in the ESD-P group and 1005 patients were in the ESD-N group. Propensity score-matching created 41 matched pairs. According to the adjusted comparisons, ESD-P required a significantly longer procedure time (85 min vs 51 min, *p* < 0.001). Other treatment outcomes showed an en bloc resection rate of 100% for both groups (*p* = 1) and complete resection rates of 95.1 and 97.6% (*p* = 1), curative resection rates of 90.2 and 90.2% (*p* = 1), perforation during ESD rates of 2.4 and 0% (*p* = 1), and postprocedure bleeding rates of 2.4 and 2.4% (*p* = 1) for the ESD-P and ESD-N groups, respectively. For the ESD-P group, lesions on the suture line or anastomotic site were significantly associated with longer procedure times (*p* = 0.038).

**Conclusions:**

ESD-P was a more time-consuming procedure than ESD-N. However, ESD-P and ESD-N achieved high rates of curative resection with a low rate of adverse events for the treatment of EGC. ESD could be selected as the treatment for EGC even in the postoperative stomach provided that careful attention is given to lesions on the suture line or anastomotic site.

## Background

Total and subtotal gastrectomies have been performed for the treatment of gastric cancer and benign peptic ulcers [1]. In addition, esophagectomy with reconstruction using a gastric tube has been used for esophageal cancer [2, 3]. However, there is a risk of gastric cancer in the postoperative stomach after surgery if total gastrectomy is not performed [2–4]. Gastric cancer in the postoperative stomach and non-operative stomachs can be detected by follow-up surveillance endoscopy [5]. Endoscopic mucosal resection and endoscopic submucosal dissection (ESD) were performed for early gastric cancer (EGC) with a negligible risk of lymph node metastasis (LNM). The indication for endoscopic resection (ER) was defined according to the current Japanese Gastric Cancer Treatment Guidelines [6]. ESD is the currently accepted treatment for lesions with absolute or expanded indications for ER. Long-term outcomes have been favorable for patients who underwent curative resection [7, 8]. Similar to ESD of the non-operative stomach (ESD-N) for EGC, ESD of the postoperative stomach (ESD-P) for EGC has been applied to and performed. Several studies reported that ESD-P is an effective and safe treatment modality for EGC and ESD-N [9–12]. Furthermore, it was reported that long-term outcomes of ESD-P for EGC were favorable when curative resection was obtained [11, 12].

However, ESD-P for EGC is known as a technically difficult procedure because of the presence of submucosal fibrosis and staples around the suture line or anastomotic site [9–13]. Some reports have shown high resection rates but with a higher risk of complications with ESD-P than with ESD-N [14, 15]. However, there were some differences in the baseline characteristics for both groups, which might have affected the outcomes. The rates of tumors in the upper third of the stomach were higher than those of the tumors in the other parts of the stomach after distal gastrectomy, which is also technically difficult [16–19]. Recently, a propensity score-matching analysis has been used to compensate for the differences in the baseline characteristics between the two groups [20–22]. Therefore, the present study aimed to examine the difficulty of ESD-P for EGC compared with ESD-N for EGC by using a propensity score-matching analysis.

## Methods

### Study design

This was a retrospective, single-center, observational cohort study. We reviewed the medical data from the ESD database, endoscopic reports, and medical records at Kitakyushu Municipal Medical Center (Fukuoka, Japan).

### Patients

ESD was performed to treat 1371 consecutive patients with gastric neoplasms in the non-operative or postoperative stomach between January 1, 2004, and July 31, 2016, at Kitakyushu Municipal Medical Center. One hundred thirty-two patients were excluded from this analysis, because two or more lesions were simultaneously resected by ESD. Furthermore, 193 patients were excluded because the resected specimen was not carcinoma, such as adenoma, according to the current Japanese classification of gastric carcinoma [23]. Therefore, 1046 patients with 1046 lesions were finally included in this study. Forty-one lesions were in the postoperative stomach, whereas 1005 lesions were in the non-operative stomach. Clinicopathological characteristics and clinical outcomes of ESD were collected from the ESD database.

### ESD procedures

All ESD procedures were performed for hospitalized patients. A total of 5–10 mg of midazolam hydrochloride and 15 mg of pentazocine hydrochloride were administered intravenously for sedation just before and during the procedure. A standard single-channel endoscope (GIF-Q260J; Olympus Optical, Tokyo, Japan) was used. VIO 300D or ICC 200 (ERBE Elektromedizin, Tubingen, Germany) was used as the power source for electrical cutting and coagulation. We performed ESD using a standard technique described elsewhere [24–26]. Briefly, marking dots were placed around the lesion using an endo-knife. To lift the lesion, a normal saline solution and hyaluronate sodium with a small amount of epinephrine (0.001 mg/mL) and 0.8% indigo carmine were injected in the submucosal layer. Then, a circumferential mucosal incision was created and submucosal dissection was performed with an endo-knife to complete the removal of the lesion. Hemostasis was achieved for bleeding or exposed vessels by using an endo-knife or hemostatic forceps. Insulated tip knife, needle-type knife, or scissor-type knife was mainly used as an endo-knife for ESD. Hook knife (KD-620; Olympus) was sometimes used during the dissection of the submucosa as a rescue device, which was useful for dissecting the fibrotic site.

### Histological evaluation

After retrieval, ESD specimens were flattened, fixed in 10% formalin, and sectioned serially at 2-mm intervals. Then, the curability of the specimens was carefully evaluated pathologically according to the Japanese Gastric Cancer Classification [23]. Resections were assessed as curative or non-curative according to the current Japanese gastric cancer treatment guidelines [6]. Non-curative resections were defined as cases that did not meet the curative criteria after ESD.

### Clinical outcomes

The primary outcome of this study was procedure time, which was defined as the time from the beginning of the mucosal incision to resection completion. A prolonged procedure was defined when completion of ESD required more than 60 min [27]. Secondary outcomes of this study were resection rates (including en bloc, complete, and curative resections) and adverse event rates (including perforation and postprocedural bleeding). A lesion resected in one piece was considered en bloc resection. A lesion resected in an en bloc pattern with tumor-free lateral and vertical margins was considered complete resection. A lesion resected in a complete resection pattern that fulfilled the curative criteria was considered curative resection. Perforations were diagnosed endoscopically during the ESD procedure or by free air during abdominal radiography or CT after ESD. Perforations detected during the ESD procedure were closed with metal clips. Postoperative bleeding was diagnosed as clinical evidence of bleeding after ESD, such as melena and hematemesis with a decrease of < 2 g/dL in the hemoglobin level or bleeding confirmed by routine or emergency second-look endoscopy that required transfusion or additional endoscopic hemostasis. We also investigated the proportion of patients who underwent ESD using multiple kinds of endo-knives in both groups.

### Statistics

This was a retrospective study without randomization. Therefore, there were potential confounding biases among the two groups involved. According to previous reports, tumor size, tumor location, depth of tumor invasion, the presence of an ulcer (scar), tumor histology, and skill of the operator were associated with the difficulty of the ESD procedure [16, 18, 19, 27–29]. To balance the bias, we used logistic regression of the following factors for the propensity score calculation [30]: age (≥75 years vs < 75 years), sex (male vs female), tumor size (≥21 mm vs < 21 mm), tumor location (in the upper third vs in the middle third or lower third of the stomach), depth of tumor invasion (mucosa vs submucosa), tumor histology (differentiated vs undifferentiated), ulcer (scar) (presence vs absence), and operator skill level (expert vs trainee). Previous studies reported that 30–60 procedures was the cut-off number for the learning curve of ESD [29, 31, 32]. Therefore, experts were defined as those with experience in performing 50 or more ESD procedures, whereas trainees were those with experience in performing fewer than 50 ESD procedures. In this study, there were 28 experts and 13 trainees. Using calipers (0.2) with a width equal to 0.25 of the standard deviation of the logit of the propensity score, we performed propensity score analysis with 1:1 matching using the nearest neighbor matching method. We estimated the area under the receiver-operating characteristics curve, which was 0.77 and thus indicated good predictive power, to validate the model of this study. We evaluated the two groups by using the absolute standardized differences (ASDs) before and after matching to confirm propensity scoring balance. If ASDs were within 1.96√2/n after matching, then we considered the characteristics to be well-balanced [33]. We analyzed the baseline characteristics and outcomes of this study using the χ^2^ or Fisher exact tests for categorial data or the Mann-Whitney U test for continuous data not distributed normally. In addition, univariate analysis was conducted to evaluate tumor factors involving long procedure times for ESD-P by using Fisher exact test for the categorical data.

*P* < 0.05 was considered statistically significant for all tests. All statistical data analyses were performed using JMP Pro 13.0 software.

### Ethics

We conducted this study in accordance with the Declaration of Helsinki. This study protocol was approved by the institutional review board of Kitakyushu Municipal Medical Center (no. 201701056). Written informed consent was obtained from all patients before treatment in accordance with the institutional protocol. The first and last authors take complete responsibility for the integrity of the data and the accuracy of the data analysis.

## Results

### Baseline characteristics before propensity score-matching

**Fig. 1:**
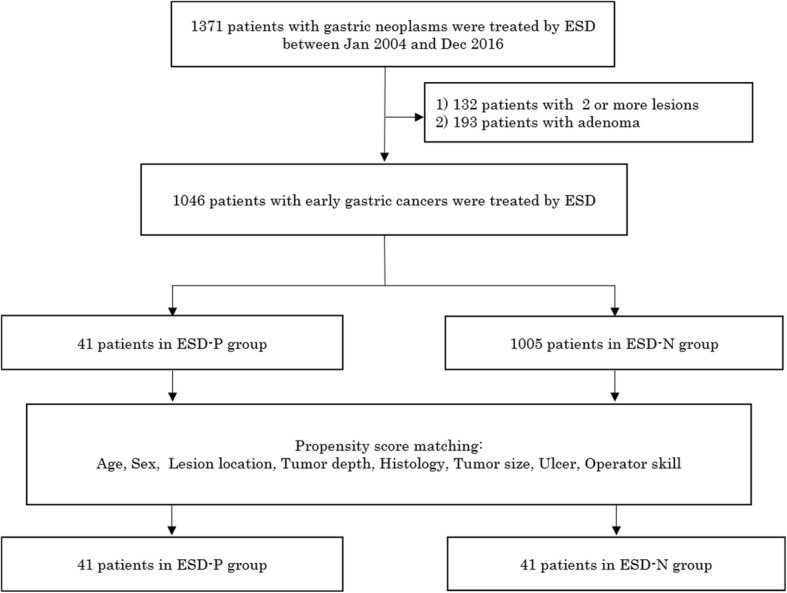
Flowchart of patients and lesions enrolled in this study. ESD, endoscopic submucosal dissection; ESD-P, endoscopic submucosal dissection of the postoperative stomach; ESD-N, endoscopic submucosal dissection of the non-operative stomach

ESD-P and ESD-N was performed in 41 and 1005 patients, respectively (shown in Fig. 1). The baseline characteristics of 1046 patients are shown in Table 1. Significantly higher percentages of male patients, those with the original tumor location in the upper third of the stomach, and experts comprised the ESD-P group. There were no significant differences among the two groups regarding other factors. The types of previous gastrectomy performed for lesions in the postoperative stomach included 27 distal gastrectomies (65.9%), 5 proximal gastrectomies (12.2%), 1 pylorus-preserving gastrectomy (2.4%), and 8 esophagectomies (19.5%).

**Table 1:** Baseline characteristics of the 1046 patients who underwent ESD before propensity score-matching

	ESD-P	ESD-N	p	ASD
	*n* = 41	*n* = 1005		
Age			0.83	
Mean ± SD	70.4 ± 9.16	71.0 ± 8.82		
Median (range)	70 (65–78)	72 (65–77)		0.834
Sex			0.004*	
Male	37 (90.2%)	706 (70.2%)		0.14
Female	4 (9.8%)	299 (29.8%)		0.14
Lesion location			< 0.001*	
U	13 (31.7%)	157 (15.6%)		0.39
M	24 (58.5%)	501 (34.5%)		0.50
L	4 (9.8%)	347 (49.9%)		0.97
Morphology			0.62	
Protruding	16 (39.1%)	322 (32.0%)		0.15
Flat	0 (0%)	11 (1.1%)		0.15
Depressed	25 (61.0%)	672 (66.9%)		0.12
Histology			0.27	
Differentiated	39 (95.1%)	981 (97.6%)		0.13
Undifferentiated	2 (4.9%)	24 (2.4%)		0.13
Lesion size (mm)			0.06	
Mean ± SD	15.0 ± 7.16	19.8 ± 13.8		0.44
Median (range)	14 (4–35)	16 (2–115)		
Specimen size (mm)			0.56	
Mean ± SD	38.7 ± 8.18	42.4 ± 15.3		0.30
Median (range)	40 (16–60)	40 (15–140)		
Depth			0.50	
pT1a	37 (90.2%)	858 (85.4%)		0.15
pT1b	4 (9.8%)	147 (14.6%)		0.15
Ulcer (scar)			0.76	
Presence	2 (4.9%)	80 (8.0%)		0.12
Absence	39 (95.1%)	921 (92.0%)		0.12
Suture line or anastomotic site			–	
Involving	19 (%)	–		–
Not involving	22 (%)	–		–
Skill level			< 0.001*	
Expert	36 (87.8%)	623 (62.0%)		0.62
Trainee	5 (12.2%)	382 (38.0%)		0.62

*ESD* endoscopic submucosal dissection, *SD* standard deviation, *ASD* absolute standardized differences, *U* upper third of the stomach, *M* middle third of the stomach, *L* lower third of the stomach, *pT1a* tumor invasion within the mucosa, *pT1b* tumor invasion in the submucosa or deeper

The *p* value was calculated using the χ^2^ test or Fisher exact test for categorical data

The *p* value was calculated using the Mann-Whitney U test for continuous data not normally distributed

*Significant

### Matched factors after propensity score-matching

Propensity score-matching created 41 matched pairs (shown in Fig. 1). All patients in the ESD-P group were matched. The matching factors of the ESD-P and ESD-N groups are shown in Table. 2. There were no significant differences in any factors among the two groups. In addition, all ASDs were within 1.96√2/n (0.43) for all matching factors. Therefore, all matched factors after propensity score-matching were well-balanced in this study.

**Table 2:** Matching factors between ESD-P and ESD-N after propensity score-matching

	ESD-P	ESD-N	P	ASD
	*n* = 41	*n* = 41		
Variable-matching between groups				
Age (y), mean ± SD	70.4 ± 9.16	72.9 ± 7.99	0.29	0.33
Age (y), median (IQR)	70 (65–78)	73 (67–80)		
Sex, male/female	37/4	38/3	1	0.15
Tumor size (mm), mean ± SD	15.0 ± 7.16	15.4 ± 9.36	0.73	0.048
Tumor depth, T1a/T1b	37/4	36/5	1	0.078
Lesion location, U/M and L	13/28	13/28	1	0
Histology, differentiated/undifferentiated	39/2	39/2	1	0
Presence of ulceration	2	2	1	0
Operator skill, expert/trainee	36/5	36/5	1	0

*ESD-P*, endoscopic submucosal dissection of the postoperative stomach, *ESD-N* endoscopic submucosal dissection of the non-operative stomach, *SD* standard deviation, *ASD* absolute standardized differences, *pT1a* tumor invasion within the mucosa, *pT1b* tumor invasion in the submucosa or deeper, *U* upper third of the stomach, *M* middle third of the stomach, *L* lower third of the stomach

The *p* value was calculated using the χ^2^ test or Fisher exact test for the categorical data

The *p* value was calculated using the Mann-Whitney U test for continuous data not normally distributed

### Treatment outcomes after propensity score-matching

The parameters of treatment outcomes are shown in Table 3. Procedure times for the ESD-P group were significantly longer than those for the ESD-N group (*P* < 0.001). The en bloc resection rate was 100% for both groups. Complete resection rates were 95.1% (39/41) for the ESD-P group and 97.6% (40/41) for the ESD-N group. The curative resection rate was 90.2% (37/41) for both groups. Regarding adverse events, the perforation rate was 2.4% (1/41) for the ESD-P group and 0% (0/41) for the ESD-N group. The postprocedural bleeding rate was 2.4% (1/41) for both groups. There were no significant differences regarding treatment outcomes, except for procedure time. However, one patient with perforation during the ESD procedure in the ESD-P group underwent emergency surgery because of postoperative peritonitis. The lesion was located on the suture line. Severe submucosal fibrosis on the suture line was detected during submucosal dissection. Although we used carbon dioxide inflation during the ESD procedure and the site of perforation was closed with metal clips immediately after the ESD procedure, the patient still had peritonitis the next day. Postoperative bleeding occurred in one patient in each group. All cases of postoperative bleeding were successfully managed by endoscopic hemostasis with hemostatic forceps; thus, blood transfusions were not required. According to the pathological evaluation, four patients in each group did not meet the curative criteria after ESD. All four patients in the ESD-P group were followed up without any additional treatment. However, one among these four patients underwent additional surgery because a residual tumor was detected in the specimen resected during the initial surgery.

**Table 3:** Treatment outcomes for ESD-P and ESD-N after propensity score-matching

	ESD-P	ESD-N	*P*
	*n* = 41	*n* = 41	
Procedure time, min, median (IQR)	85 (57–120)	51 (28–79)	< 0.001*
Involving suture line, min, median (IQR)	120.0 (89.0–160.0)	–	
Not involving suture line, min, median (IQR)	60.5 (38.5–84.75)	–	
Procedure time, min, mean ± SD	95.0 ± 59.0	57.8 ± 39.4	
Long procedure time	30 (73.2%)	16 (39.0%)	0.004*
En bloc resection, n (%)	41 (100%)	41 (100%)	–
Complete resection, n (%)	39 (95.1%)	40 (97.6%)	1
Curative resection, n (%)	37 (90.2%)	37 (90.2%)	1
Perforation, n (%)	1 (2.4%)	0 (0%)	1
Postprocedure bleeding, n (%)	1 (2.4%)	1 (2.4%)	1
Use of multiple kinds of endo-knives, n(%)	15 (36.6%)	7 (17.1%)	0.080

ESD-P, endoscopic submucosal dissection of the postoperative stomach; ESD-N, endoscopic submucosal dissection of the non-operative stomach; SD, standard deviation; IQR, interquartile range

The p value was calculated using the χ^2^ test or Fisher exact test for categorical data

The p value was calculated using the Mann-Whitney U test for continuous data not normally distributed

*Significant value

In addition, the proportion of patients who underwent ESD-P using multiple kinds of endo-knives was higher than that of patients who underwent ESD-N using multiple kinds of endo-knives, but not significant (36.6% in ESD-P vs 17.1 in ESD-P, *P* = 0.080, shown in Table 3).

Table 4 shows the factors associated with prolonged procedures in the ESD-P group. Tumors involving the suture line or anastomotic site were significantly associated with prolonged procedures (*p* = 0.038), but previous surgery type, size, depth, location, morphology, histology, and presence of an ulcer (scar) in the tumor were not significantly associated with prolonged procedures.

**Table 4:** Tumor factors involving long procedure times for ESD-P

	Long procedure time	Shorter procedure time	P
	*n* = 30	*n* = 11	
Previous surgery type, (%)			0.412
Distal gastrectomy	20 (66.7%)	7 (63.6%)	
Proximal gastrectomy	3 (10.0%)	2 (18.2%)	
Pylorus preserving gastrectomy	0 (0%)	1 (9.1%)	
Esophagectomy	7 (23.3%)	1 (9.1%)	
Tumor size, n (%)			0.651
< 21	24 (80%)	10 (90.9%)	
≥ 21	6 (20%)	1 (9.1%)	
Tumor depth, n (%)			0.559
T1a	26 (86.7%)	11 (100%)	
T1b	4 (13.3%)	0 (0%)	
Lesion location, n (%)			0.719
Upper	9 (30.0%)	4 (36.4%)	
Middle or lower	21 (70.0%)	7 (63.6%)	
Suture line or anastomotic site, n (%)			0.038*
Involved	17 (56.7%)	2 (18.2%)	
Not involved	13 (43.4%)	9 (81.8%)	
Morphology, n (%)			0.287
Flat or depressed	20 (66.7%)	5 (45.5%)	
Protruding	10 (33.3%)	6 (54.5%)	
Histology, n (%)			1
Differentiated	28 (93.3%)	11 (100%)	
Undifferentiated	2 (6.7%)	0 (0%)	
Ulceration (scar)			1
Presence	2 (6.7%)	0 (0%)	
Absence	28 (93.3%)	11 (100%)	

*ESD-P* endoscopic submucosal dissection for early gastric cancer in the postoperative stomach

The p value was calculated using the χ^2^ test or the Fisher exact test for categorical data

*Significant

## Discussion

To the best of our knowledge, this is the first study comparing the clinical outcomes between ESD-P and ESD-N for the treatment of EGC using a propensity score-matching analysis. Additionally, various types of post-operative stomachs, including not only distal gastrectomy but also proximal gastrectomy, pylorus preserving gastrectomy and esophagectomy, were included and assessed in the present study. Although a high rate of curative resection with a low rate of adverse event for ESD was achieved regardless of the type of post-operative stomach, ESD-P for EGC required significantly longer procedure times than ESD-N for EGC. Moreover, one patient who underwent ESD-P for EGC only on the suture line required emergency surgery.

In this study, 65.9% (27/41) of the patients in the ESD-P group who underwent distal gastrectomy required resection of the middle and lower thirds of the stomach, indicating that EGC of ESD-P frequently originally developed in the upper third of the stomach. It is generally accepted that it is more difficult to complete ESD in the upper third than in the middle third or lower third of the stomach [16–19]. Therefore, it is not appropriate to directly compare the clinical outcomes between ESD-P and ESD-N, because the lesion locations in both procedures significantly differ; thus, we performed a propensity score-matching analysis and included this factor as a covariate-matching factor to compensate for this bias. Therefore, we were able to compare the treatment outcomes for both groups after controlling for potential sources of bias according to the covariates. The tumor location and other covariates did not affect the treatment outcomes of ESD for both groups.

Lesions on the suture line or anastomotic site involve the submucosal fibrosis [9, 13, 15]; therefore, performing ESD for these lesions was difficult to complete. In the present study, 17 lesions had developed on the suture line of the postoperative stomach, whereas 2 lesions on the anastomotic site. Even though ESD of these lesions was performed only by experts, the median ESD time was significantly longer for these lesions than for lesions not involving the suture line or anastomotic site (120.0 min vs 60.5 min; *P* < 0.001). There was no significant difference in the procedure time between lesions on the suture line and those on the anastomotic site (120.0 min vs 130 min; *P* = 0.69). Lesion development on the suture line or anastomotic site was the only independent factor associated with prolonged procedure times (Table 4). In addition, it has been reported that ESD of lesions on the suture line is associated with a high rate of perforation [9, 15]. In this study, there was only one perforation case during ESD of a lesion located on the suture line in the postoperative stomach, even though experts performed the ESD procedure. Despite the non-negligible risk of perforation and prolonged procedure time, a high rate of curative resection has been achieved, even for lesions that developed on the suture line or anastomotic site of the postoperative stomach. The complication rate of ESD-P in the present study was lower than those of previous reports [9–12]. This might be because of the fact that 87.8% (36/41) of ESD-P procedures were performed by experts. ESD-P should be performed cautiously by highly experienced experts, especially when the lesions are located on the suture line or anastomotic site.

We should take the difference in cost of ESD between the groups into account in addition to the procedure time. The proportion of patients who underwent ESD-P using multiple kinds of endo-knives seems to be higher than that of patients who underwent ESD-N using multiple kinds of endo-knives, although it did not reach to a statistical significance (36.6% in ESD-P vs 17.1% in ESD-N; *P* = 0.080, shown in Table 3). Therefore, it seemed that ESD-P was costlier than ESD-N in terms of the number of devices used during the procedure.

In this study, consistent with previous reports [9–12], high rates of en bloc and complete resections and resulting high rates of curative resection were achieved not only in the ESD-N group but also in the ESD-P group. All patients with curative resection in both groups had no recurrence. In contrast, there were four patients with non-curative resection in each group. Regarding ESD-N, one of the four patients with non-curative resection underwent additional surgery, which resulted in finding a remnant tumor of the resected specimen. The remaining patients did not undergo any additional treatments, and all had no recurrence. According to the current guidelines [6], additional surgery is indicated for all patients with non-curative ESD resection and significant risk for LNM. However, it has been reported that almost half of the EGC patients with non-curative ESD resection did not undergo additional surgery due to their advanced age or comorbidities [34]. Furthermore, it has been reported that LNM was found in only 5–10% of patients with such lesions in the non-operative stomach [35]. Regarding ESD-P, all four patients who underwent non-curative ESD resection were followed up without any additional treatments. The risk of LNM in T1 remnant cancer after distal gastrectomy resection was reported to be only 2.4% (1/42) [36]. Patients who underwent non-curative ESD resection in the remnant stomach refused radical completion gastrectomy of the remnant stomach due to the high risk of complications and postoperative mortality rates (from 13 to 41%) [37–40]. Completion gastrectomy for EGC of the remnant stomach is much riskier during the perioperative period compared to that of the non-remnant stomach [37–40]. Given the few data regarding the prognosis of patients with non-curative ESD resection for the postoperative stomach, it should be considered whether additional surgery should be performed based on the current guidelines due to the non-negligible risk of postoperative mortality for radical completion gastrectomy of the remnant stomach. As a result, all four patients with non-curative ESD resection in the ESD-P group had no recurrence. A recent report suggested a useful scoring system for evaluating the risk of LNM for non-curative patients [41]. Using this scoring system, 3 patients who underwent non-curative ESD-P were classified as low-risk patients, with an estimated LNM risk of 2–3%. The other patient was classified as having an intermediate risk, with estimated LNM risk of 6–7%. In this study, there were no high-risk patients. This system may be useful for deciding additional surgery even for patients who underwent non-curative ESD-P. However, some lymph nodes might be resected in patients who underwent the previous surgery for a malignant lesion. This system may not be applicable to non-curative patients with post-operative stomach. In the future, further expanded criteria or more appropriate risk stratifications may be required for ESD-P for EGC.

This study had some limitations. First, this was a single-center retrospective study with a relatively small sample size. Second, we did not consider the use of traction method, such as a traction method using dental floss and a hemoclip, which was recently reported to shorten the procedure time of ESD [42]. Third, we did not consider medications, especially anti-thrombotic drugs, in this study, since they were discontinued during the perioperative period, because the patients enrolled in this study had undergone ESD treatments before the current Japanese guidelines for the use of anti-thrombotic agents were established [43]. Hence, there are no data regarding the influence of anti-thrombotic agents on ESD-P. Fourth, we could not deny the possibility that the learning curve might affect the technical outcome of this present study, which could not be fully compensated by the propensity score-matching. Fifth, ESD-P was performed by only 9 experts, specifically 8 experts who performed ESD-P involving the suture line or anastomotic site, whereas, 8 experts performed ESD-N after propensity score-matching. Seven experts were in common among ESD-P and ESD-N after matching Furthermore, 6 experts were in common among ESD-P involving suture line or anastomic site and ESD-N after matching. Therefore, there seems to be some bias of operators. Sixth, we did not consider the difference of the electrosurgical units (VIO300D or ICC200) used in the ESD procedures, which might have affected the study outcome. A larger prospective, randomized, controlled trial will be required to determine further treatment guidelines, including the influence of anti-thrombotic agents, for ESD-P.

## Conclusion

In conclusion, ESD-P was a more time-consuming procedure than ESD-N. However, ESD-P and ESD-N for the treatment of EGC achieved high rates of curative resection with low rates of adverse events. Therefore, ESD could be selected as the treatment for EGC, even in the postoperative stomach, but careful attention must be focused on lesions on the suture line or anastomotic site.

## Abbreviations

ASD: Absolute standardized differences
CT: Computed tomography
EGC: Early gastric cancer
ER: Endoscopic resection
ESD: Endoscopic submucosal dissection
ESD-N: Endoscopic submucosal dissection of the non-operative stomach
ESD-P: Endoscopic submucosal dissection of the postoperative stomach
LNM: Lymph node metastasis
